# Supplementary material for: The causal relationship between the human gut microbiota and pyogenic arthritis: a Mendelian randomization study
Source: Front Cell Infect Microbiol. 2024 Nov 26;14:1452480. doi: 10.3389/fcimb.2024.1452480 (PMC11629706; doi:10.3389/fcimb.2024.1452480)
Supplement: Supplementary file 1 [file DataSheet1.zip › mendelian test/ebi-a-GCST90027649.csv_forest.pdf]

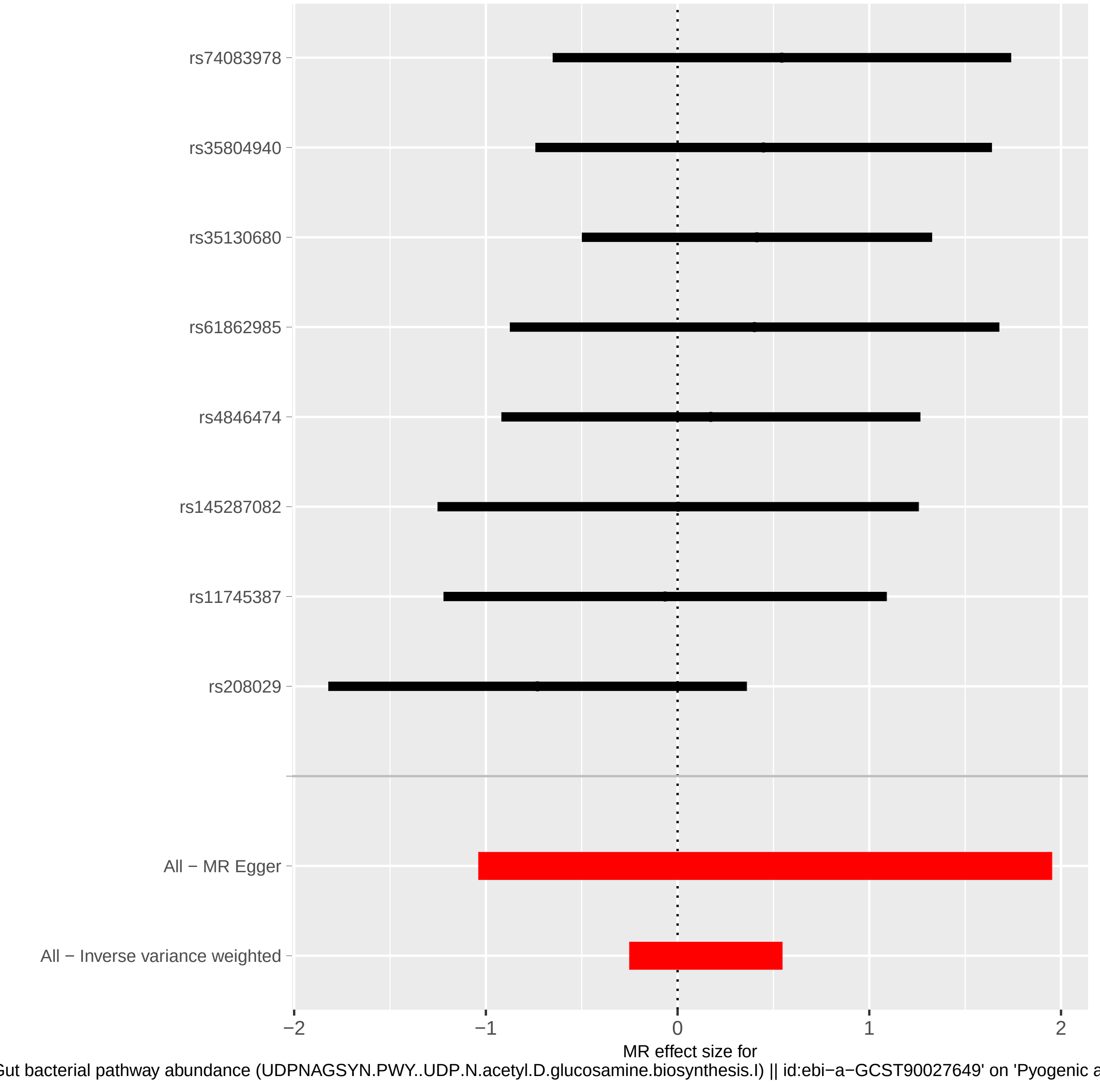

rs74083978
rs35804940
rs35130680
rs61862985
rs4846474
rs145287082
rs11745387
rs208029
All − MR Egger
All − Inverse variance weighted
−2
−1
0
1
2
MR effect size for
Gut bacterial pathway abundance (UDPNAGSYN.PWY..UDP.N.acetyl.D.glucosamine.biosynthesis.I) || id:ebi−a−GCST90027649' on 'Pyogenic a
